# Helmholtz: A Verifier for Tezos Smart Contracts Based on Refinement Types

**DOI:** 10.1007/978-3-030-72013-1_14

**Published:** 2021-02-26

**Authors:** Yuki Nishida, Hiromasa Saito, Ran Chen, Akira Kawata, Jun Furuse, Kohei Suenaga, Atsushi Igarashi

**Affiliations:** 8 Eindhoven University of Technology, Eindhoven, The Netherlands; 9 Aalborg University, Aalborg East, Denmark; 10 Kyoto University, Kyoto, Japan; 11 DaiLambda, Inc., Kyoto, Japan

## Abstract

A *smart contract* is a program executed on a blockchain, based on which many cryptocurrencies are implemented, and is being used for automating transactions. Due to the large amount of money that smart contracts deal with, there is a surging demand for a method that can statically and formally verify them.

This tool paper describes our type-based static verification tool Helmholtz for Michelson, which is a statically typed stack-based language for writing smart contracts that are executed on the blockchain platform Tezos. Helmholtz is designed on top of our extension of Michelson’s type system with refinement types. Helmholtz takes a Michelson program annotated with a user-defined specification written in the form of a refinement type as input; it then typechecks the program against the specification based on the refinement type system, discharging the generated verification conditions with the SMT solver Z3. We briefly introduce our refinement type system for the core calculus Mini-Michelson of Michelson, which incorporates the characteristic features such as compound datatypes (e.g., lists and pairs), higher-order functions, and invocation of another contract. Helmholtz successfully verifies several practical Michelson programs, including one that transfers money to an account and that checks a digital signature.

## 1 Introduction

A *blockchain* is a data structure to implement a distributed ledger in a trustless yet secure way. The idea of blockchains is initially devised for the Bitcoin cryptocurrency [12] platform. Many cryptocurrencies are implemented using blockchains, in which value equivalent to a significant amount of money is exchanged.

Recently, many cryptocurrency platforms allow programs to be executed on a blockchain. Such programs are called *smart contracts* [19] (or, simply a *contract* in this paper) since they work as a device to enable automated execution of a contract. In general, a smart contract is a program $P_a$ associated with an account

⋆ Current affiliation: Preferred Networks, Inc.

$a$ on a blockchain. When the account $a$ receives money from another account $b$ with a parameter $v$, the computation defined in $P_a$ is conducted, during which the state of the account $a$ (e.g., the balance of the account and values that are stored by the previous invocations of $P_a$) which is recorded on the blockchain may be updated. The contract $P_a$ may execute money transactions to another account (say $c$), which results in invocations of other contracts (say $P_c$) during or after the computation; therefore, contract invocations may be chained.

Although smart contracts' original motivation was handling simple transactions (e.g., money transfer) among the accounts on a blockchain, recent contracts are being used for more complicated purposes (e.g., establishing a fund involving multiple accounts). Following this trend, the languages for writing smart contracts also evolve from those that allow a contract to execute relatively simple transactions (e.g., Script for Bitcoin) to those that allow a program that is as complex as one written in standard programming languages (e.g., EVM for Ethereum and Michelson [1] for Tezos [4]).

Due to a large amount of money they deal with, verification of smart contracts is imperative. *Static* verification is especially needed since a smart contract cannot be fixed once deployed on a blockchain. Attack on a vulnerable contract indeed happened. For example, the DAO attack, in which the vulnerability of a fundraising contract was exploited, resulted in the loss of cryptocurrency equivalent to approximately 150M USD [18].

In this paper, we describe our type-based static verifier HELMHOLTZ[3] for smart contracts written in Michelson. The Michelson language is a statically- and simply typed stack-based language equipped with rich data types (e.g., lists, maps, and higher-order functions) and primitives to manipulate them. Although several high-level languages that compile to Michelson are being developed, Michelson is most widely used to write a smart contract for Tezos as of writing.

A Michelson program expresses the above computation in a purely functional style, in which the Michelson program corresponding to $P_a$ is defined as a function. The function takes a pair of the parameter $v$ and a value $s$ that represents the current state of the account (called *storage*) and returns a pair of a list of *operations* and the updated storage $s'$. Here, an operation is a Michelson value that expresses the computation (e.g., transferring money to an account and invoking the contract associated with the account) that is to be conducted after the current computation (i.e., $P_a$) terminates. After the computation specified by $P_a$ finishes with a pair of a storage value and an operation list, a blockchain system invokes the computation specified in the operation list. This purely functional style admits static verification methods for Michelson programs similar to those for standard functional languages.

As the theoretical foundation of HELMHOLTZ, we design a refinement type system for Michelson as an extension of the original simple type system. In contrast to standard refinement types that refine the types of values, our type

[3] Hermann von Helmholtz (1821–1894), a German physicist and physician, was a doctoral advisor of Albert A. Michelson (1852–1931), whom the Michelson language is apparently named after.

system refines the type of *stacks*. We briefly describe our type system in Section 3; a detailed explanation is deferred to a future paper.

We show that our tool can verify several practical smart contracts. In addition to the contracts we wrote ourselves, we apply our tool to the sample Michelson programs used in Mi-cho-coq [3], a formalization of Michelson in Coq proof assistant [21]. These contracts consist of practical contracts such as one that checks a digital signature and one that transfers money.

We note that Helmholtz currently supports approximately 80% of the whole instructions of the Michelson language. Another limitation of the current Helmholtz is that it can verify only a single contract, although one often uses multiple contracts for an application, in which a contract may call another by a money transfer operation, and their behavior as a whole is of interest. We are currently extending Helmholtz so that it can deal with more programs.

Our contribution is summarized as follows: (1) Definition of the core calculus Mini-Michelson and its refinement type system; (2) Automated verification tool Helmholtz for Michelson contracts implemented based on the type system of Mini-Michelson; the interface to the implementation can be found at https://www.fos.kuis.kyoto-u.ac.jp/trylang/Helmholtz; and (3) Evaluation of Helmholtz with various Michelson contracts, including practical ones.

The rest of this paper is organized as follows. Before introducing the technical details, we present an overview of the verifier Helmholtz in Section 2 using a simple example of a Michelson contract. Section 3 introduces the core calculus Mini-Michelson and its refinement type system. Section 4 describes the verifier Helmholtz, a case study, and experimental results. After discussing related work in Section 5, we conclude in Section 6.

## 2 Overview of Helmholtz and Michelson

We overview our tool Helmholtz in this section before presenting its technical details. We also explain Michelson by example (Section 2.2) and user-written annotation added to a Michelson program for verification purposes (Section 2.3).

### 2.1 Helmholtz

As input, Helmholtz takes a Michelson program annotated with (1) its specification expressed in a refinement type and (2) additional user annotations such as loop invariants. It typechecks the annotated program against the specification using our refinement type system; the verification conditions generated during the typechecking is discharged by the SMT solver Z3 [11]. If the code successfully typechecks, then the program is guaranteed to satisfy the specification.

Helmholtz is implemented as a subcommand of `tezos-client`, the client program of the Tezos blockchain. For example, to verify `boomerang.tz` in Figure 1, we run `tezos-client refinement boomerang.tz`. If the verification succeeds, the command outputs `VERIFIED` to the terminal screen (with a few log messages); otherwise, it outputs `UNVERIFIED`.

```
1  parameter unit;
2  storage unit;
3  << ContractAnnot { (param, st) | True } ->
4     { (ops, st') | amount = 0 && ops = [] ||
5        amount <> 0 && ops = [Transfer Unit amount (Contract source)]}
6     & { _ | False } >>
7  code                     /* (param,st) */
8   { CDR;                  /* st */
9     NIL operation;        /* [] ▷ st */
10    AMOUNT;               /* amount ▷ [] ▷ st */
11    PUSH mutez 0;         /* 0 ▷ amount ▷ [] ▷ st */
12    IFCMPEQ
13     {                    /* [] ▷ st   (amount ≤ 0) */ }
14     {                    /* [] ▷ st   (amount > 0) */
15       SOURCE;            /* src ▷ [] ▷ st */
16       CONTRACT unit;     /* Some (Contract src) ▷ [] ▷ st */
17       ASSERT_SOME;       /* (Contract src) ▷ [] ▷ st */
18       AMOUNT; UNIT;      /* Unit ▷ amount ▷ (Contract src) ▷ [] ▷ st */
19       TRANSFER_TOKENS;   /* Transfer Unit amount (Contract src) ▷ [] ▷ st */
20       CONS               /* [Transfer Unit amount (Contract src)] ▷ st */
21     };
22            /* ops ▷ st, where ops is the top element at the end of each branch, namely,
23               [Transfer Unit amount (Contract src)] if amount > 0; or [] otherwise */
24    PAIR    /* (ops, st) */
25  }
```

**Fig. 1.** `boomerang.tz`. The comment inside `/* */` describes the stack at the program point.

## 2.2 An Example Contract in Michelson

Figure 1 shows an example of a Michelson program called `boomerang`. A Michelson program is associated with an account on the Tezos blockchain; the program is invoked by transferring money to this account. This artificial program in Figure 1, when it is invoked, is supposed to transfer the received money back to the account that initiated the transaction.

A Michelson program starts with type declarations of its `parameter`, whose value is given by contract invocation, and `storage`, which is the state that the contract account stores. Lines 1–2 declare that the types of both are `unit`, the type inhabited by the only value `Unit`. Lines 3–6 surrounded by `<<` and `>>` are a user-written annotation used by HELMHOLTZ for verification; we will explain this annotation later. The `code` section in Lines 8–24 is the body of this program.

Let us take a look at the `code` section of the program. In the following explanation of each instruction, we describe the state of the stack after each instruction as comments; stack elements are delimited by ▷.

– Execution of a Michelson program starts with a stack with one value, which is a pair `(param, st)` of a parameter `param` and a storage value `storage`.
– `CDR` pops the pair at the top of the stack and pushes the second value of the popped pair; therefore, after executing the instruction, the stack contains the single value `st`.
– `NIL` pushes the empty list `[]` to the stack; the instruction is accompanied by the type `operation` of the list elements for typechecking purposes.

– AMOUNT pushes the nonnegative amount of the money sent to the account to which this program is associated.
– PUSH mutez 0 pushes the value 0. The type mutez represents a unit of money used in Tezos.
– IFCMPEQ b1 b2, if the state of the stack before executing the instruction is v1 ▷ v2 ▷ tl, (1) pops v1 and v2 and (2) executes the then-branch b1 (resp., the else-branch b2) if v2 = v1 (resp., v2 ≠ v1). In boomerang, this instruction does nothing if amount = 0; otherwise, the instructions in the else-branch are executed.
– SOURCE at the beginning of the else-branch pushes the address src of the source account, which initiated the chain of contract invocations that the current contract belongs to, resulting in the stack src ▷ [] ▷ st.
– CONTRACT $T$ pops an address addr from the stack and typechecks whether the contract associated with addr takes an argument of type $T$. If the typechecking succeeds, then Some (Contract addr) is pushed; otherwise, None is pushed. The constructor Contract creates an object that represents a typechecked contract at the given address. In Tezos, the source account is always a contract that takes the value Unit as a parameter; thus, Some (Contract src) will always be pushed onto the stack.
– ASSERT_SOME pops a value v from the stack and pushes v' if v is Some v'; otherwise, it raises an exception.
– UNIT pushes the unit value Unit to the stack.
– TRANSFER_TOKENS, if the stack is of the shape varg ▷ vamt ▷ vcontr ▷ tl, pops varg, vamt, and vcontr from the stack and pushes (Transfer varg vamt vcontr) onto tl. The value Transfer varg vamt vcontr is an *operation object* expressing that money (of amount vamt) shall be sent to the account vcontr with the argument varg after this program finishes without raising an exception. Therefore, the program associated with vcontr is invoked after this program finishes.
– CONS with the stack v1 ▷ v2 ▷ tl pops v1 and v2, and pushes a cons list v1::v2 onto the stack. (We use the list notation in OCaml here.)
– After executing one of the branches associated with IFCMPEQ in this program, the shape of the stack should be ops ▷ storage, where ops is [] if amount = 0 or [Transfer varg vamt vcontr] if amount > 0. The instruction PAIR pops ops and storage, and pushes (ops,storage).

A Michelson program is supposed to finish its execution with a singleton stack whose unique element is a pair of (1) a list of operations to be executed after the current execution of the contract finishes and (2) the new value for the storage.

Michelson is a statically typed language. Each instruction is associated with a typing rule that specifies the shapes of stacks before and after it by a sequence of simple types such as int and int list. For example, CONS requires the type of top element to be $T$ and that of the second to be $T$ list (for any $T$); it ensures the top element after it has type $T$ list.

Other notable features of Michelson include first-class functions, hashing, instructions related to cryptography such as signature verification, and manipulation of a blockchain using operations.

### 2.3 Specification

A user can specify the behavior of a program by a `ContractAnnot` annotation, which is a part of the augmented syntax of our verification tool. A `ContractAnnot` annotation gives a specification of a Michelson program by the following notation inspired by the refinement types: {`(param,st) | pre`} `->` {`(ops,st')` `| post`} `&` {`exc | abpost`} where `pre`, `post`, and `abpost` are predicates. This specification reads as follows: if this program is invoked with a parameter `param` and storage `st` that satisfies the property `pre`, then (1) if the execution of this program succeeds, then it returns a list of operations `ops` and new storage `storage'` that satisfy the property `post`; (2) if this program raises an exception with value `exc`, then `exc` satisfies `abpost`. The specification language is expressive enough to cover the specifications for practical contracts, including the ones we used in the experiments in Section 4.3. In the predicates, one can use several keywords such as `amount` for the amount of the money sent to this program when it is invoked and `source` for the source account's address.

The `ContractAnnot` annotation in Figure 1 (Lines 3–6) formalizes this program's specification as follows. This program can take any parameter and storage (Line 3). Successful execution of this program results in a pair `(ops,st')` that satisfies the condition in Lines 4–5 that expresses (1) if `amount` $= 0$, then `ops` is empty, that is, no operation will be issued; (2) if `amount` $> 0$, then `ops` is a list of a single element `Transfer Unit amount (Contract source)`, which expresses transfer of money of the amount `amount` to the account at `source` with the unit argument.[4] In the specification language, `source` and `amount` are keywords that stand for the source account and the amount of money sent to this program, respectively. The part `& { _ | False }` expresses that this program does not raise an exception. This specification correctly formalizes the intended behavior of this program.

## 3 Refinement Type System for Mini-Michelson

In this section, we formalize Mini-Michelson, a core subset of Michelson with its syntax, operational semantics, and refinement type system. We also state that the type system is sound. We omit many features from the full language in favor of conciseness but includes language constructs—such as higher-order functions and iterations—that make verification difficult.

Figure 2 shows the syntax of Mini-Michelson. *Values*, ranged over by $V$, consist of integers $i$; addresses $a$; operations `transaction` $(V, i, a)$ to invoke a contract at $a$ by sending money of amount $i$ and an argument $V$; pairs $(V_1, V_2)$ of values; the empty list $[\,]$; cons $V_1 :: V_2$; and code $\langle IS \rangle$ of first-class functions.[5]

[4] As we mentioned in Section 1, HELMHOLTZ can currently verify the behavior of a single contract, although there will be an invocation of the contract associated with `source` after the termination of `boomerang`. An operation is treated as an opaque data structure, from which one cannot extract values.

[5] Closures are not needed because functions in Michelson can access only arguments.

$$
\begin{array}{rcl}
V & ::= & i \mid a \mid \texttt{transaction}(V, i, a) \mid (V_1, V_2) \mid [\,] \mid V_1 :: V_2 \mid \langle IS \rangle \\
T & ::= & \texttt{int} \mid \texttt{address} \mid \texttt{operation} \mid T_1 \times T_2 \mid T\,\texttt{list} \mid T_1 \to T_2 \\
IS & ::= & \{I_1; \ldots; I_n\} \\
I & ::= & IS \mid \texttt{DIP}\,IS \mid \texttt{DROP} \mid \texttt{DUP} \mid \texttt{SWAP} \mid \texttt{PUSH}\,T\,V \mid \texttt{NOT} \mid \texttt{ADD} \mid \texttt{IF}\,IS_1\,IS_2 \mid \\
 & & \texttt{LOOP}\,IS \mid \texttt{PAIR} \mid \texttt{CAR} \mid \texttt{CDR} \mid \texttt{NIL}\,T \mid \texttt{CONS} \mid \texttt{IF\_CONS}\,IS_1\,IS_2 \mid \texttt{ITER}\,IS \mid \\
 & & \texttt{LAMBDA}\,T_1\,T_2\,IS \mid \texttt{EXEC} \mid \texttt{TRANSFER\_TOKENS}\,T
\end{array}
$$

**Fig. 2.** Syntax of Mini-Michelson

Unlike Michelson, we use integers as a substitute for Boolean values so that 0 means *false* and the others mean *true*. *Simple types*, ranged over by $T$, consist of base types (`int`, `address`, and `operation`, which are self-explanatory), pair types $T_1 \times T_2$, list types $T\,\texttt{list}$, and function types $T_1 \to T_2$. *Instruction sequences*, ranged over by $IS$, are a sequence of *instructions*, ranged over by $I$, enclosed by curly braces. A Mini-Michelson *program* is an instruction sequence.

Instructions include those for stack manipulation (to `DROP`, `DUP`licate, `SWAP`, and `PUSH` values); `NOT` and `ADD` for manipulating integers; `PAIR`, `CAR`, and `CDR` for pairs; `NIL` and `CONS` for constructing lists; and `TRANSFER_TOKENS` to create an operation that expresses a money transfer after the current contract execution. The instruction `IF` branches depending on whether the stack top is 0 or not; `IF_CONS` branches on whether the stack top is a cons or not. The instruction `LOOP` $IS$ repeats $IS$ as long as the stack top is a nonzero integer at the loop entry; `ITER` $IS$ is for iterating the list at the stack top. `LAMBDA` pushes a function (described by its operand $IS$) onto the stack, and `EXEC` calls a function. Perhaps unfamiliar is `DIP` $IS$, which pops and saves the stack top somewhere else, executes $IS$, and then pushes the saved value back.

We also use a few kinds of stacks in the following definitions: value stacks, ranged over by $S$, type stacks, ranged over by $\bar{T}$, and type binding stacks, ranged over by $\Upsilon$, of the form $x_1 : T_1 \triangleright .. \triangleright x_n : T_n$. The empty stack is denoted by $\ddagger$, and push is by $\triangleright$. We often omit the empty stack and write, for example, $V_1 \triangleright V_2$ for $V_1 \triangleright V_2 \triangleright \ddagger$. Intuitively, $T_1 \triangleright .. \triangleright T_n$ and $x_1 : T_1 \triangleright .. \triangleright x_n : T_n$ describe stacks $V_1 \triangleright .. \triangleright V_n$ where each value $V_i$ is of type $T_i$. We will use variables to name stack elements in the refinement type system.

Mini-Michelson (as well as Michelson) is equipped with a simple type system. The type judgment for instructions is written $\bar{T} \vdash I \Rightarrow \bar{T}'$, which means that instruction $I$ transforms a stack of type $\bar{T}$ into another stack of type $\bar{T}'$. The type judgment for values is written $V : T$, which means that $V$ is given simple type $T$. We omit typing rules as they are fairly straightforward.

### 3.1 Operational Semantics

We give a big-step operational semantics of Mini-Michelson by defining the judgment $S \vdash I \Downarrow S'$, which means that executing the instruction $I$ under the stack $S$ results in the stack $S'$, (and also $S \vdash IS \Downarrow S'$). Most rules for $S \vdash I \Downarrow S'$ are straightforward. We show rules for `DIP` and `LOOP` below and omit other rules.

$$\frac{S \vdash IS \Downarrow S'}{V \rhd S \vdash \texttt{DIP}\, IS \Downarrow V \rhd S'} \qquad \frac{S \vdash IS \Downarrow S' \quad S' \vdash \texttt{LOOP}\, IS \Downarrow S'' \quad (i \neq 0)}{i \rhd S \vdash \texttt{LOOP}\, IS \Downarrow S''} \qquad \frac{}{0 \rhd S \vdash \texttt{LOOP}\, IS \Downarrow S}$$

The first rule means that the body *IS* is executed with the stack $S$ obtained by removing the top element $V$, which is pushed back onto the resulting stack $S'$. There are two rules for `LOOP`: the first rule means that if the stack top is nonzero, then the body is executed, and then the execution of `LOOP` *IS* is repeated; the second rule means that, if the stack top is zero, then the loop acts as a no-op.

### 3.2 Refinement Type System

In the refinement type system, a simple stack type $T_1 \rhd .. \rhd T_n$ is augmented with a formula $\varphi$ of first-order logic to describe the relationship among stack elements. We introduce *refinement stack types*, ranged over by $\Phi$, of the form $\{x_1 : T_1 \rhd ... \rhd x_n : T_n \mid \varphi(x_1, ..., x_n)\}$, which denotes stacks $V_1 \rhd .. \rhd V_n$ such that $V_1 : T_1, \ldots, V_n : T_n$ and $\varphi(V_1, ..., V_n)$ hold.

We show (part of) the syntax of terms and formulae of the first-order logic:

$$t ::= x \mid V \mid \texttt{transaction}\,(t_1, t_2, t_3) \mid t_1 :: t_2 \mid (t_1, t_2) \mid t_1 + t_2 \mid \cdots$$
$$\varphi ::= t_1 = t_2 \mid \texttt{call}\,(t_1, t_2) = t_3 \mid \varphi_1 \vee \varphi_2 \mid \neg\, \varphi \mid \exists\, x : T.\varphi \mid \cdots$$

The language for predicates is multi-sorted, where a sort is a simple type of Michelson. The sorting rules for term constructors and relation symbols are standard. For example, in $t_1 + t_2$, both $t_1$ and $t_2$ have to be of sorts `int`; and in $t_1 = t_2$, the sorts of $t_1$ and $t_2$ must be the same, and so on. The only relation symbol worth explaining is $\texttt{call}\,(t_1, t_2) = t_3$, which informally means that calling function $t_1$ with argument $t_2$ (as the only element of the input stack) yields a stack consisting only of $t_3$ as a result. We use other predicates, connectives, and quantifiers such as $t_1 \neq t_2$, $\varphi_1 \wedge \varphi_{12}$, $\varphi_1 \implies \varphi_2$, and $\forall\, x : T.\varphi$, which can be considered as derived forms.

We define the semantics of the formulae in a standard manner. Let $\sigma$ be a *value assignment*, i.e., a sort-respecting finite map from variables to values. We define the interpretation $[\![t]\!]_\sigma$ of $t$ under $\sigma$ and *valid* formulae under a value assignment, denoted by $\sigma \models \varphi$; for $\texttt{call}\,(t_1, t_2) = t_3$, we define $\sigma \models \texttt{call}\,(t_1, t_2) = t_3$ iff $[\![t_2]\!]_\sigma \rhd \ddagger \vdash [\![t_1]\!]_\sigma \Downarrow [\![t_3]\!]_\sigma \rhd \ddagger$. Equality on instruction sequences is intensional: formula $\langle IS \rangle = \langle IS' \rangle$ is valid only if *IS* and *IS'* are syntactically equal.

For a finite mapping $\Gamma$ (called a type environment) from variables to sorts, $\Gamma \models \sigma$ and $\Gamma \models \varphi$ are defined as usual: $\Gamma \models \sigma$ iff $\texttt{dom}\,(\sigma) = \texttt{dom}\,(\Gamma)$ and $\sigma(x) : \Gamma(x)$ for any $x \in \texttt{dom}\,(\sigma)$; $\Gamma \models \varphi$ iff $\sigma \models \varphi$ for any value assignment $\sigma$ such that $\Gamma \models \sigma$.

The type system is equipped with subtyping whose judgment is of the form $\Gamma \vdash \Phi_1 <: \Phi_2$, which means stack type $\Phi_1$ is a subtype of $\Phi_2$ under $\Gamma$. The type judgment for instructions (resp. instruction sequences) is of the form $\Gamma \vdash \Phi_1\ I\ \Phi_2$ (resp. $\Gamma \vdash \Phi_1\ IS\ \Phi_2$), which means that, under $\Gamma$, if $I$ (resp. *IS*) is executed under a stack satisfying $\Phi_1$, the resulting stack (if the execution terminates) satisfies $\Phi_2$. We often call $\Phi_1$ *pre-condition* and $\Phi_2$ *post-condition*.

We show representative typing rules in Figure 3.

$$\frac{\Gamma, x : T \vdash \{\Upsilon \mid \varphi\}\ \mathit{IS}\ \{\Upsilon' \mid \varphi'\}}{\Gamma \vdash \{x : T \triangleright \Upsilon \mid \varphi\}\ \texttt{DIP}\ \mathit{IS}\ \{x : T \triangleright \Upsilon' \mid \varphi'\}} \quad \text{(RT-Dip)}$$

$$\frac{\Gamma \vdash \{\Upsilon \mid \exists\, x : \texttt{int}.\varphi\ \wedge\ x \neq 0\}\ \mathit{IS}_1\ \Phi \qquad \Gamma \vdash \{\Upsilon \mid \exists\, x : \texttt{int}.\varphi\ \wedge\ x = 0\}\ \mathit{IS}_2\ \Phi}{\Gamma \vdash \{x : \texttt{int} \triangleright \Upsilon \mid \varphi\}\ \texttt{IF}\ \mathit{IS}_1\ \mathit{IS}_2\ \Phi} \quad \text{(RT-If)}$$

$$\frac{\Gamma \vdash \{\Upsilon \mid \exists\, x : \texttt{int}.\varphi\ \wedge\ x \neq 0\}\ \mathit{IS}\ \{x : \texttt{int} \triangleright \Upsilon \mid \varphi\}}{\Gamma \vdash \{x : \texttt{int} \triangleright \Upsilon \mid \varphi\}\ \texttt{LOOP}\ \mathit{IS}\ \{\Upsilon \mid \exists\, x : \texttt{int}.\varphi\ \wedge\ x = 0\}} \quad \text{(RT-Loop)}$$

$$\frac{y_1' : T_1 \vdash \{y_1 : T_1 \mid y_1' = y_1\ \wedge\ \varphi_1\}\ \mathit{IS}\ \{y_2 : T_2 \mid \varphi_2\}}{\Gamma \vdash \{\Upsilon \mid \varphi\}\ \texttt{LAMBDA}\ T_1\ T_2\ \mathit{IS}\ \{x : T_1 \to T_2 \triangleright \Upsilon \mid \varphi\ \wedge\ \forall\, y_1' : T_1, y_2 : T_2.\varphi_1[y_1 := y_1']\ \wedge\ \texttt{call}\,(x, y_1') = y_2\ \Longrightarrow\ \varphi_2\}} \quad \text{(RT-Lambda)}$$

$$\frac{}{\Gamma \vdash \{x_1 : T_1 \triangleright x_2 : T_1 \to T_2 \triangleright \Upsilon \mid \varphi\}\ \texttt{EXEC}\ \{x_3 : T_2 \triangleright \Upsilon \mid \exists\, x_1 : T_1, x_2 : T_1 \to T_2.\varphi \wedge \texttt{call}\,(x_2, x_1) = x_3\}} \quad \text{(RT-Exec)}$$

$$\frac{\Gamma \vdash \Phi_1 <: \Phi_1' \qquad \Gamma \vdash \Phi_1'\ I\ \Phi_2' \qquad \Gamma \vdash \Phi_2' <: \Phi_2}{\Gamma \vdash \Phi_1\ I\ \Phi_2} \quad \text{(RT-Sub)}$$

**Fig. 3.** Typing rules (excerpt)

- (RT-Dip) means that DIP $\mathit{IS}$ is well typed if the body $\mathit{IS}$ is typed under the stack type obtained by removing the top element. The popped value named $x$ is moved to the type environment part so that it can be referred to in the refinement predicate $\varphi$ in the pre-condition.
- (RT-If) means that the instruction is well typed if both branches have the same post-condition; the pre-conditions of the branches are strengthened by the assumptions that the top of the input stack is true ($x \neq 0$) and false ($x = 0$). The variable $x$ is existentially quantified because the top element will be removed before the execution of either branch.
- (RT-Loop) is similar to the proof rule for while-loops in Hoare logic. The formula $\varphi$ is a loop invariant. Since the body of LOOP is executed while the stack top is nonzero, the pre-condition for the body $\mathit{IS}$ is strengthened by $x \neq 0$, whereas the post-condition of LOOP $\mathit{IS}$ is strengthened by $x = 0$.
- (RT-Lambda) is for the instruction to push a first-class function onto the operand stack. The premise of the rule means that the body $\mathit{IS}$ takes a value (named $y_1$) of type $T_1$ that satisfies $\varphi_1$ and outputs a value (named $y_2$) of type $T_2$ that satisfies $\varphi_2$ (if it terminates). The post-condition in the conclusion expresses, by using call, that the function $x$ has the property above. The extra variable $y_1'$ in the type environment of the premise is an alias of $y_1$; being a variable declared in the type environment $y_1'$ can appear in both $\varphi_1$ and $\varphi_2$[6] and can describe the relationship between the input and output of the function.
- (RT-Exec) adds $\texttt{call}\,(x_2, x_1) = x_3$ to the post-condition, meaning that the result of a call to the function $x_2$ with $x_1$ as an argument yields $x_3$. It may look simpler than expected; the crux here is that $\varphi$ is expected to imply $\forall\, x_1 : T_1, x_3 : T_2.\varphi_1 \wedge \texttt{call}\,(x_2, x_1) = x_3\ \Longrightarrow\ \varphi_2$, where $\varphi_1$ and $\varphi_2$ represent

[6] The scope of a variable in a refinement stack type is its predicate part and so $y_1$ cannot appear in the post-condition.

the pre- and post-conditions, respectively, of function $x_2$. If $x_1$ satisfies $\varphi_1$, then we can derive that $\varphi_2$ holds.

– (RT-SUB) is the rule for subsumption to strengthening the pre-condition and weakening the post-condition. In our type system, subtyping is defined semantically: A *subtyping* judgment $\Gamma \vdash \{\Upsilon \mid \varphi_1\} <: \{\Upsilon \mid \varphi_2\}$ holds if for any $\sigma$ such that $\forall x \in \mathtt{dom}\,(\Gamma, \Upsilon).\sigma(x) : (\Gamma, \Upsilon)(x)$, $\sigma \models \varphi_1 \implies \varphi_2$ is valid. (Here, by abuse of notation, the type binding stack $\Upsilon$ is regarded as a mapping from variables to sorts.)

We state that our type system is *sound*: For a well-typed instruction, if we execute the instruction under a stack that satisfies the pre-condition of the typing, then (if the execution halts) the resulting stack satisfies the post-condition of the typing. To state the soundness theorem, we define an auxiliary relation $\Gamma \models S : \Phi$, which means "stack $S$ satisfies stack refinement type $\Phi$ under environment $\Gamma$", by: $\Gamma \models V_1 \triangleright .. \triangleright V_m : \{y_1 : T_1' \triangleright .. \triangleright y_m : T_m' \mid \varphi\} \iff V_1 : T_1', \ldots, V_m : T_m'$ and $\sigma[y_1 \mapsto V_1, .., y_m \mapsto V_m] \models \varphi$ for any $\sigma$ such that $\Gamma \models \sigma$.

Then, the soundness theorem, whose proof will appear in a forthcoming full version, is stated as follows:

**Theorem 1 (Soundness).** *If $\Gamma \vdash \Phi_1$ IS $\Phi_2$, $\Gamma \models S : \Phi_1$, and $S \vdash IS \Downarrow S'$, then $\Gamma \models S' : \Phi_2$.*

**Sketch of Typechecking** We implement a typechecking algorithm as follows. Given a type environment, a pre-condition, and a post-condition, our algorithm computes the strongest post-condition of the code starting from the given pre-condition. This computation is conducted according to the syntax-directed version of the typing rules created essentially in the same way as a type system with subtyping (e.g., one described in [15]). An application of the subtyping generates verification conditions. The accumulated verification conditions are fed to Z3; the typechecking succeeds if they are successfully discharged.

### 3.3 Extensions

The implementation supports a few extensions of the formalization explained above, which are explained below.

The type system implemented in HELMHOLTZ is extended with refinements for values thrown by raising exceptions. For example, the typing rule for instruction `FAILWITH`, which raises an exception with the value at the stack top, is given as follows:

$$\Gamma \vdash \{x : T \triangleright \Upsilon \mid \varphi\}\ \mathtt{FAILWITH}\ \{\Upsilon \mid \bot\}\&\{\mathtt{err} \mid \exists x : T, \Upsilon.\varphi \land x = \mathtt{err}\}.$$

The rule expresses that, if `FAILWITH` is executed under a non-empty stack that satisfies $\varphi$, then the program point just after the instruction is not reachable (hence, $\{\Upsilon \mid \bot\}$). The refinement $\exists x : T, \Upsilon.\varphi \land x = \mathtt{err}$ for the exception case states that $\varphi$ in the pre-condition with the top element $x$ is equal to the raised value `err`; since $x$ is not in the scope in the exception refinement, $x$ is bound by an existential quantifier. The typing rules for the other instructions can be extended with the "&" part easily.

Helmholtz deals with measure functions introduced by Kawaguchi et al. [9] and supported by Liquid Haskell [23]. If a measure function is defined by a `Measure` annotation, Helmholtz "weaves" the function definition into relevant typing rules. For instance, given the annotation `Measure len : list int -> int where [] = 0 | h :: t = (1 + len t)`, Helmholtz assumes an uninterpreted function symbol `len` and augments (RT-Nil) and (RT-Cons) as follows, where the last equality in each post-condition comes from the definition of `len`.

$$\Gamma \vdash \{\Upsilon \mid \varphi\}\ \texttt{NIL}\, T\ \{x : T\,\texttt{list} \triangleright \Upsilon \mid \varphi \wedge x = [\,] \wedge \texttt{len}\,[\,] = 0\}$$

$$\Gamma \vdash \{x_1 : T \triangleright x_2 : T\,\texttt{list} \triangleright \Upsilon \mid \varphi\}\ \texttt{CONS}\ \{x_3 : T\,\texttt{list} \triangleright \Upsilon \mid \exists\, x_1 : T, x_2 : T\,\texttt{list}.\varphi \wedge x_1 :: x_2 = x_3 \wedge \texttt{len}\,(x_1 :: x_2) = 1 + \texttt{len}\,x_2\}$$

## 4 Tool Implementation

In this section, we discuss annotations in detail, show a case study of contract verification, and present verification experiments.

### 4.1 Annotations

Helmholtz supports several forms of annotations (surrounded by `<<` and `>>` in the source code), other than `ContractAnnot` explained in Section 2.

`Assert` $\Phi$ and `Assume` $\Phi$ can appear before or after an instruction. The former asserts that the stack at the annotated program location satisfies the type $\Phi$; the assertion is verified by Helmholtz. If there is an annotation `Assume` $\Phi$, Helmholtz assumes that the stack satisfies the type $\Phi$ at the annotated program location. A user can give a hint to Helmholtz by using `Assume` $\Phi$. The user has to make sure that it is correct; if an `Assume` annotation is incorrect, the verification result may be incorrect.

`LoopInv` $\Phi$ asserts the loop invariant of a loop instruction (e.g., `LOOP` and `ITER`). In the current implementation, annotating a loop invariant using `LoopInv` $\Phi$ is mandatory. Helmholtz checks that $\Phi$ is indeed a loop invariant and uses it to verify the rest of the program.

In the current implementation, a `LAMBDA` instruction, which pushes a function on the top of the stack, must be accompanied by the `LambdaAnnot` annotation, where $\Phi_{\mathrm{pre}} \to \Phi_{\mathrm{post}}\ \&\ \Phi_{\mathrm{abpost}}$ is a specification of the pushed function and the bindings $(x_1 : T_1, \ldots, x_n : T_n)$ introduce the ghost variables that can be used in the annotations in the body of the annotated `LAMBDA` instruction;[7] one can omit the declaration of ghost variables if it is empty. The first contract in Figure 4, which pushes a function that takes a pair of integers and returns the sum of them, presents an example of `LambdaAnnot`. The annotated type of the function (Line 5)

[7] `ContractAnnot` also allows declarations of ghost variable used in the `code` section.

```
1  parameter unit ;
2  storage int;
3  << ContractAnnot { _ | True } -> { _ | True } & { _ | False } >>
4  code { DROP;
5         << LambdaAnnot { p | p = (3, 1) } -> { x | x = 4 } & { _ | False }
6            (a:int, b:int) >>
7         LAMBDA (pair int int) int
8          { << Assume { p | p = (a, b) } >>
9            UNPAIR; ADD
10           << Assert { p | p = a + b } >>
11         };
12        PUSH int 1; PUSH int 3; PAIR; EXEC;
13        << Assert { x | x = 4 } >>
14        NIL operation; PAIR
15      }
```

```
parameter (list int);
storage int;
<< Measure len : list int -> int where [] = 0 | h :: t = (1 + len t) >>
<< ContractAnnot
   { (p, _) | True } -> { (_, ret) | len p = ret } & { _ | False } >>
code { CAR; PUSH int 0; SWAP;
       << LoopInv { l : n | len l + n = len p } >>
       ITER { DROP; PUSH int 1; ADD };
       NIL operation;
       PAIR
     }
```

**Fig. 4.** `lambda.tz`, which uses higher-order functions, and `length.tz`, which uses a measure function in the contract annotation.

expresses that it returns 4 if it is fed with a pair $(3, 1)$. The ghost variables $a$ and $b$ are used in the annotations `Assume` (Line 8) and `Assert` (Line 10) in the body to denote the first and the second arguments of the pair passed to this function.

HELMHOLTZ allows user-defined (recursive) functions to be used in annotations; these functions are called *measure functions* following the terminology of Liquid-Haskell [9]. The annotation `Measure` $x : T_1 \to T_2$ `where` $p_1 = e_1 \mid \cdots \mid p_n = e_n$ defines a recursive function $x$ that takes a value of type $T_1$, destructs it by the pattern matching, and returns a value of type $T_2$. Metavariables $p$ and $e$ represent ML-like patterns and expressions. The second contract in Figure 4, which computes the length of the list passed as a parameter, exemplifies the usage of the `Measure` annotation. This contract defines a measure function `len` that takes a list of integers and returns its type; it is used in `ContractAnnot` and `LoopInv`.

## 4.2 Case Study: Contract with Signature Verification

Figure 5 presents the code of the contract `checksig.tz`, which verifies that a sender indeed signed certain data using her private key. This contract uses instruction `CHECK_SIGNATURE`, which is supposed to be executed under a stack of the form `key` $\triangleright$ `sig` $\triangleright$ `bytes` $\triangleright$ `tl`, where `key` is a public key, `sig` is a signature, and `bytes` is some data. `CHECK_SIGNATURE` pops these three values from the

```
parameter (pair signature string);
storage (pair address key);
<< ContractAnnot
   { (param, store) | match Contract store.first with
                        Contract<string> _ -> True | _ -> False } ->
   { (ops, new_store) | store = new_store &&
        sig store.second param.first (Pack param.second) &&
        ops = [ Transfer param.second 1 (Contract store.first) ] }
   & { _ | not (sig store.second param.first (Pack param.second)) } >>
code  { DUP; DUP; DUP;
        DIP { CAR; UNPAIR; DIP { PACK } }; CDDR;
        CHECK_SIGNATURE; ASSERT;
        UNPAIR; CDR; SWAP; CAR;
        CONTRACT string; ASSERT_SOME; SWAP;
        PUSH mutez 1; SWAP;
        TRANSFER_TOKENS;
        NIL operation; SWAP;
        CONS; DIP { CDR };
        PAIR
      }
```

**Fig. 5.** `checksig.tz`, which involves signature verification.

stack and pushes `true` if `sig` is the valid signature for `bytes` with the private key corresponding to `key`.

The intended behavior of `checksig.tz` is as follows. It stores a pair of an address `addr`, which is the address of a contract that takes a `string` parameter, and a public key `key` in its storage. It takes a pair `(sig,s)` of type `pair signature string` as a parameter where `signature` is the primitive Michelson type for signatures. This contract terminates without exception if `sig` is created from the serialized (packed) representation of `s` and signed by the private key corresponding to `key`. In a normal termination, this contract transfers 1 `mutez` to the contract with address `addr`. If this signature verification fails, then an exception is raised.

This behavior is expressed as a specification in the `ContractAnnot` annotation in `checksig.tz` as follows.

- The refinement of its pre-condition part expresses that the address stored in the first element `store.first` of the storage `store` is an address of a contract that takes a value of type `string` as a parameter. This is expressed by the pattern-matching of `Contract store.first`, which represents the contract stored at the address `store.first`, to the pattern expression `Contract<string> _`, which matches a contract that takes a `string` value.
- The refinement of the post-condition forces the following three conditions: (1) the store is not updated by this contract (`store = new_store`); (2) `param.first` is the signature created from the packed string `Pack param.second` of the string in the second element of the parameter and signed by the private key corresponding to the second element `store.second` of the store (`sig store.second param.first (Pack param.second)`); and (3) the operations `ops` returned by this contract is `[ Transfer param.second 1`

`(Contract store.first) ]`, which represents an operation of transferring 1 `mutez` to the contract `Contract store.first` with the parameter `param.second`. The predicate `sig` and the constructor `Pack` are primitives of HELMHOLTZ that can be used in an annotation.
- The refinement in the exception part expresses that if an exception is raised, then the signature verification should have failed (`not (sig store.second param.first (Pack param.second))`).

HELMHOLTZ successfully verifies `checksig.tz` without any additional annotation in the `code` section. If we change the instruction `ASSERT` in Line 12 to `DROP` to let the contract drop the result of the signature verification (hence, an exception is not raised even if the signature verification fails), the verification fails as intended.

### 4.3 Experiments

We applied HELMHOLTZ to various contracts; Table 1 is an excerpt of the result, in which we show (1) the number of the instructions in each contract (column #instr.) and (2) time (ms) spent to verify each contract. The experiments are conducted on MacOS Catalina 10.15.7 with Dual-Core Intel Core i5 (1.8 GHz), 8 GB RAM. We used Z3 version 4.8.8. The contracts `boomerang.tz`, `deposit.tz`, `manager.tz`, `vote.tz`, and `reservoir.tz` are taken from the benchmark of Mi-cho-coq [3]. `checksig.tz` is derived from `weather_insurance.tz` of the official Tezos test suite.[8] `vote_for_delegate.tz` and `xcat.tz` are taken from the official test suite; `xcat.tz` is simplified from the original. `triangular_num.tz` is a simple test case that we made as an example of using `LOOP`. The source code of these contracts can be found at the Web interface of HELMHOLTZ. Each contract is supposed to work as follows.

- `boomerang.tz`: Transfers the received amount of money to the source account.
- `deposit.tz`: Transfers money to the sender if the address of the sender is identical to that is stored in the storage.
- `manager.tz`: Calls the passed function if the address of the caller matches the address stored in the storage.
- `vote.tz`: Accepts a vote to a candidate if the voter transfers enough voting fee, and stores the tally.
- `checksig.tz`: The one explained in Section 4.2.
- `vote_for_delegate.tz`: Delegates one's ballot in voting by stakeholders, which is one of the fundamental features of Tezos, to another using a primitive operation of Tezos.
- `xcat.tz`: Transfers all stored money to one of the two accounts specified beforehand if called with the correct password. The account that gets money is decided based on whether the contract is called before or after a deadline.

[8] https://gitlab.com/tezos/tezos/-/tree/ee2f75bb941522acbcf6d5065a9f3b2/tests_python/contracts/mini_scenarios

- reservoir.tz: Sends a certain amount of money to either a contract or another depending on whether the contract is executed before or after the deadline.
- triangular_num.tz: Calculates the sum from 1 to $n$, which is the passed parameter.

In the experiments, we verified that each contract indeed works according to the intention explained above. triangular_num.tz was the only contract that required a manual annotation for verification in the code section; we needed to specify a loop invariant in this contract.

**Table 1.** Benchmark result

| Filename | #instr. | time (ms) | Filename | #instr. | time (ms) |
|---|---|---|---|---|---|
| boomerang.tz | 17 | 35 | checksig_unverified.tz | 36 | 62 |
| deposit.tz | 24 | 54 | vote_for_delegate.tz | 87 | 143 |
| manager.tz | 29 | 60 | xcat.tz | 64 | 188 |
| vote.tz | 24 | 62 | reservoir.tz | 45 | 87 |
| checksig.tz | 38 | 65 | triangular_num.tz | 16 | 35 |

Although the numbers of instructions in these contracts are not large, they capture essential features of smart contracts; everyone except triangular_num.tz executes transactions; deposit.tz and manager.tz check the identity of the caller; and checksig.tz conducts signature verification. The time spent on verification is small.

## 5 Related Work

There are several publications on the formalization of programming languages for writing smart contracts. Hirai [7] formalizes EVM, a low-level smart contract language of Ethereum and its implementation, using Lem [13], a language to specify semantic definitions; definitions written in Lem can be compiled into definitions in Coq, HOL4, and Isabelle/HOL. Based on the generated definition, he verifies several properties of Ethereum smart contracts using Isabelle/HOL. Bernardo et al. [3] implemented Mi-Cho-Coq, a formalization of the semantics of Michelson using the Coq proof assistant. They also verified several Michelson contracts. Compared to their approach, we aim to develop an automated verification tool for smart contracts. Park et al. [14] developed a formal verification tool for EVM by using the K-framework [17], which can be used to derive a symbolic model checker from a formally specified language semantics (in this case, formalized EVM semantics [6]), and successfully applied the derived model checker to a few EVM contracts. It would be interesting to formalize the semantics of Michelson in the K-framework to compare Helmholtz with the derived model checker.

The DAO attack [18], mentioned in Section 1, is one of the notorious attacks on a smart contract. It exploits a vulnerability of a smart contract that is related to a callback. Grossman et al. [5] proposed a type-based technique to verify that execution of a smart contract that may contain callbacks is equivalent to another execution without any callback. This property, called *effectively callback freedom*, can be seen as one of the criteria for execution of a smart contract not to be vulnerable to the DAO-like attack. Their type system focuses on verifying the ECF property of *execution* of a smart contract, whereas ours concerns the verification of generic functional properties of a smart contract.

Benton proposes a program logic for a minimal stack-based programming language [2]. His program logic can give an assertion to a stack as our stack refinement types do. However, his language does not support first-class functions nor instructions for dealing with smart contracts (e.g., signature verification).

Our type system is an extension of the Michelson type system with refinement types, which have been successfully applied to various programming languages [16,22,9,10,20,26,23,24,25]. DTAL [25] is a notable example of an application of refinement types to an assembly language, a low-level language like Michelson. A DTAL program defines a computation using registers; we are not aware of refinement types for stack-based languages like Michelson.

We notice the resemblance between our type system and a program logic for PCF proposed by Honda and Yoshida [8], although the targets of verification are different. Their logic supports a judgment of the form $A \vdash e :_u B$, where $e$ is a PCF program, $A$ is a pre-condition assertion, $B$ is a post-condition assertion, and $u$ represents the value that $e$ evaluates to and can be used in $B$, which resembles our type judgment in the formalization in Section 3. Their assertion language also incorporates a term expression $f \bullet x$, which expresses the value resulting from the application of $f$ to $x$; this expression resembles the formula $\texttt{call}\,(t_1, t_2) = t_3$ used in a refinement predicate. We have not noticed an automated verifier implemented based on their logic. Further comparison is interesting future work.

## 6 Conclusion

We described our automated verification tool HELMHOLTZ for the smart contract language Michelson based on the refinement type system for Mini-Michelson. HELMHOLTZ verifies whether a Michelson program follows a specification given in the form of a refinement type. We also demonstrated that HELMHOLTZ successfully verifies various practical Michelson contracts.

Currently, HELMHOLTZ supports approximately 80% of the whole instructions of the Michelson language. The definition of a measure function is limited in the sense that, for example, it can define only a function with one argument. We are currently extending HELMHOLTZ so that it can deal with more programs.

HELMHOLTZ currently verifies the behavior of a single contract, although a blockchain application often consists of multiple contracts in which contract calls are chained. To verify such an application as a whole, we plan to extend HELMHOLTZ so that it can verify an inter-contract behavior compositionally by combining the verification results of each contract.
